# Comparative Analysis of Genome Editors Efficiency on a Model of Mice Zygotes Microinjection

**DOI:** 10.3390/ijms221910221

**Published:** 2021-09-23

**Authors:** Olga A. Averina, Oleg A. Permyakov, Olga O. Grigorieva, Alexey S. Starshin, Alexander M. Mazur, Egor B. Prokhortchouk, Olga A. Dontsova, Petr V. Sergiev

**Affiliations:** 1 Institute of Functional Genomics, Lomonosov Moscow State University, 119991 Moscow, Russia; averina.olga.msu@gmail.com (O.A.A.); norad_m@mail.ru (O.A.P.); grig_forever@mail.ru (O.O.G.); 2 Belozersky Institute of Physico-Chemical Biology, Lomonosov Moscow State University, 119899 Moscow, Russia; 3 Research Center of Biotechnology of the Russian Academy of Sciences, Institute of Bioengineering, 119071 Moscow, Russia; starshin.alexey@gmail.com (A.S.S.); mazur.am@gmail.com (A.M.M.); prokhortchouk@gmail.com (E.B.P.); 4 Department of Chemistry, Lomonosov Moscow State University, 119991 Moscow, Russia; olga.a.dontsova@gmail.com; 5 Center of Life Sciences, Skolkovo Institute of Science and Technology, 121205 Moscow, Russia; 6 Shemyakin-Ovchinnikov Institute of Bioorganic Chemistry, 117997 Moscow, Russia

**Keywords:** genome editing, CRISPR Cas9, knockout mice, microinjection, homologous recombination, prime editor

## Abstract

Genome editing is an indispensable tool for functional genomics. The caveat of the genome-editing pipeline is a prevalence of error-prone non-homologous end joining over homologous recombination, while only the latter is suitable to introduce particularly desired genetic variants. To overcome this problem, a toolbox of genome engineering was appended by a variety of improved instruments. In this work, we compared the efficiency of a number of recently suggested improved systems for genome editing applied to the same genome regions on a murine zygote model via microinjection. As a result, we observed that homologous recombination utilizing an ssDNA template following sgRNA directed Cas9 cleavage is still the method of choice for the creation of animals with precise genome alterations.

## 1. Introduction

Genome editing became an indispensable tool for functional genomics of model animals [1] and livestock [2]. At the same time, it paved the way for gene therapeutics in humans [3]. The breakthrough in the field of genome editing was associated with the development of a toolbox for the modification of mammalian genomes [4,5] based on the CRISPR/Cas9 immune system of bacteria. CRISPR/Cas9 directed cleavage of a particular site of a genome results in a double-strand break, which is repaired via non-homologous end joining (NHEJ) or homologous recombination (HR). The major outcome of NHEJ is the generation of small insertions and deletions, which are frequently applied for gene inactivation via coding frame disruption. The frequencies of particular mutations generated by NHEJ might be predicted, but in general, NHEJ results in a range of mutations and might not be used for the introduction of a particular desired allelic variant. In contrast, repair by HR leads to a generation of a particular sequence variant at a site of cleavage, which might be programmed if the recombination machinery is artificially supplied with a suitable DNA template sharing homology with the DNA sequence flanking the cleavage site. The caveat of the genome-editing pipeline is a prevalence of NHEJ over HR, which is suitable for gene inactivation, problematic for the creation of a laboratory animal of a particular genotype and restrictive for potential applications in human gene therapy. To overcome this problem, a toolbox of genome engineering was appended by a set of improved instruments. The prime editor system makes use of a fusion between Cas9 H840A mutant possessing nickase instead of the double strands cleavage activity and optimized reverse transcriptase [6]. After the introduction of a nick, the resulting 3′-end is used as a primer for reverse transcription of a hybrid pegRNA molecule containing a sgRNA part followed by a template region containing a mutation intended to be incorporated into the genome. After the reannealing of the product of reverse transcription with the non-edited DNA strand and consequent repair, the desired mutation has a chance to be accepted. The absence of a double-strand break intermediate should minimize repair via error-prone NHEJ mechanisms. Another suggested approach is an application of a fusion between Cas9 and monomeric streptavidin (mSA), which might be used together with a biotinylated DNA template for HR. In this case, a biotinylated DNA template for homologous recombination is co-localized with the DNA double-strand break site via interaction with mSA. In this work, we decided to compare the efficiency of a number of recently suggested improved systems for genome editing applied to the same genome regions on a model of modification of mice zygotes by microinjection. 

## 2. Results and Discussion

**Figure 1:**
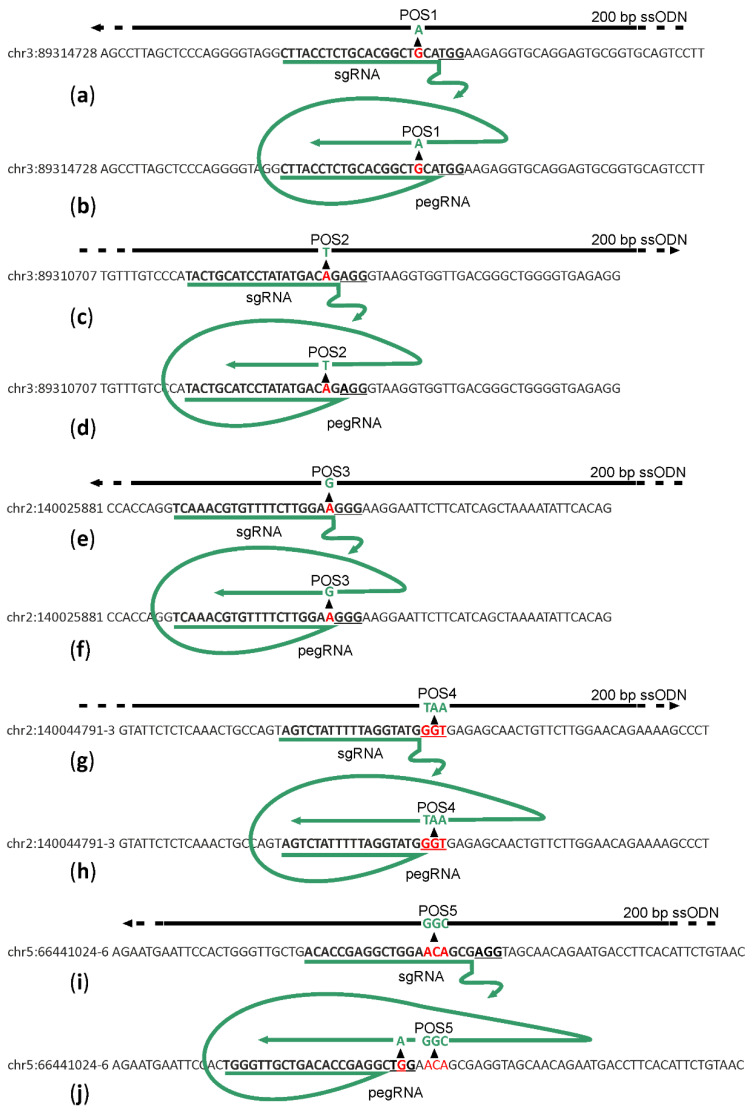
Scheme of sgRNA or pegRNA (green) for target (letters) cleavage and reverse transcription and DNA templates (black lines) for HR. (**a**,**c**,**e**,**g**,**i**) Schemes for genome regions 1–5 editing by sgRNA directed cleavage and ssDNA directed HR. (**b**,**d**,**f**,**h**,**j**) Schemes for genome regions 1–5 editing by pegRNA directed cleavage and reverse transcription. PAM sites are underlined. Red and green nucleotides mark the wild type and edited sequences, correspondingly. Editing positions are numbered as POS1–5.

To compare the efficiency of prime editor 2 (PE2) [6] with that of single-stranded DNA oligonucleotide (ssDNA) directed HR [7], we aimed to introduce base substitutions into genome positions chr3:89314728G/A (1), chr3:89310707T/A (2), chr2:140025881T/C (3), chr2:140044791-3GGT/TAA (4), chr5:66441024-6TGT/GCC (5) (Figure 1) in mice zygotes. Mouse genome assembly GRCm39 was used for genetic coordinate numbers. Substitutions corresponding to positions 1 and 2 localize to *FLAD1* gene coding for flavin adenine dinucleotide synthase whose mutations are detected in human patients with lipid storage myopathy [8]. Mutations introduced into positions 3 and 4 correspond to *NDUFAF5* gene mutations detected in human patients suffering from mitochondrial complex I deficiency [9]. Finally, position 5 is localized in the *NSUN7* gene, whose mutant forms are found in males suffering from infertility [10].

For each position, we created a set of sgRNA and 200 nt long ssDNA templates for HR centered at the desired mutation site (Figure 1a,c,e,g,i; Supplementary Table S1) and pegRNA for the originally described [6] variant of PE2 editor to introduce the desired mutations (Figure 1b,d,f,h,j; Supplementary Table S1). The sequences of sgRNAs and pegRNAs were designed to minimize the distance between the cleavage and intended mutation sites and to avoid re-cleavage of the DNA following successful mutagenesis. Since we were limited in sgRNA choice by the positions of intended mutations, we have no possibility to select sgRNAs with particularly high efficiency and selectivity scores. Where possible, we used the same sequences for sgRNA and pegRNA directed cleavage.

Particularly, cleavage and editing sites for positions 1 and 2 were designed in such a way that successful editing would result in a single base substitution at −3 position relative to the PAM. Editing of position 3 would result in substitution at position −1 to the PAM. Since this region is the most sensitive to the complementarity between sgRNA and the DNA, we expected that the introduction of designed mutations would prevent re-cleavage of the genomic DNA even in the case of position 3, where a mutation creates a new PAM sequence shifted by one nucleotide to the region of sgRNA/pegRNA complementarity. The editing site at position 4 is located within the PAM, and thus, successful editing would certainly prevent genome re-cleavage. Editing of position 5 includes 3-bp substitution at positions −4 to −6, relative to the PAM in the case of HR editing. The same cleavage site was found not to be suitable for PE2 directed editing since the site of editing should be located downstream of the nick generated by PE2. To overcome this problem, we applied another cleavage site and a designed pegRNA template region to introduce an additional substitution into the PAM site.

**Figure 2:**
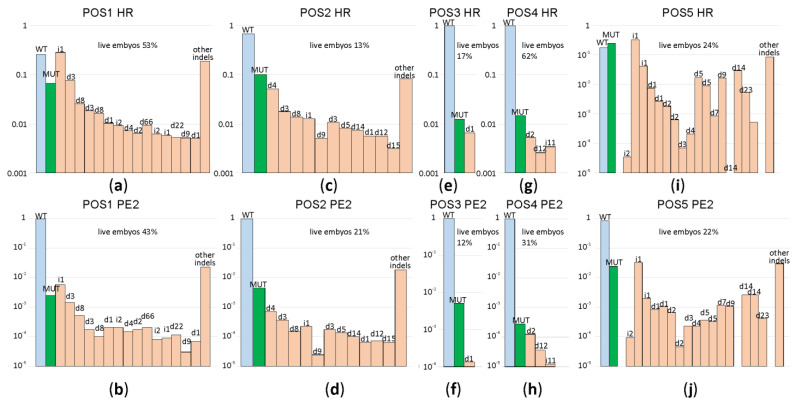
Comparison of genome editing efficiencies for ssDNA guided HR and PE2 assessed for a set of genome regions 1–5. Shown are frequencies of allelic variants, in the logarithmic scale, detected in the blastocysts developed from the zygotes after microinjection. Blue bars correspond to the wild type genotype, green to the mutation programmed by a template, while pale red to insertions and deletions. Allelic variants are designated above the bars. Panels (**a**,**c**,**e**,**g**,**i**) correspond to ssDNA guided HR, while panels (**b**,**d**,**f**,**h**,**j**) to PE2 driven genome editing. Panels (**a**,**b**) correspond to genome position 1, (**c**,**d**) to genome position 2, (**e**,**f**) to genome position 3, (**g**,**h**) to genome position 4, while (**i**,**j**) corresponds to genome position 5. For each construct, the percentage of zygotes developed to the blastocyst or morula stage is indicated above the graph.

In total, 1237 zygotes were microinjected with ssDNA, mRNA Cas9 and sgRNA (191 developed to blastocyst and 113 to morula stage) and 828 zygotes were microinjected with mRNA PE2 and pegRNA (155 developed to blastocyst and 33 to morula stage). The survival rate for each microinjection experiment can be found in Supplementary Table S2. Generally, the rates of zygotes survival and successful development to morula and blastocyst stages after microinjections were comparable for HR and PE2 genome editing, the latter being slightly lower (Figure 2). In some cases, e.g., editing of position 3, and to a lower extent, positions 2 and 5, the survival was markedly lower for both genome editing tools, which might be explained by a phenotypic consequence of the target or hypothetically off-target editing. In other favorable, cases the rate of successful development after microinjection was comparable with that for intact zygotes, which were not injected (ca. 70%). 

A subset of blastocysts (see Supplementary Table S2 for exact numbers) was used for genome DNA extraction, target DNA region amplification (Supplementary Figure S1) and Illumina sequencing. The obtained reads were aligned to the target genome regions and classified by the mutations found (Figure 2; see Supplementary Tables S3–S7 for exact sequences and frequencies of the resulting allelic variants). From the obtained result, the prime editor efficiency is low in this experimental setup, corroborating the results obtained by other groups [11]. While we observed very high variability in the editing efficiency depending on a particular genome region, for both PE2 and ssDNA directed editing, in all cases, the efficiency of the prime editor was lower than that of ssDNA directed editing. Both genome editing strategies generated indels more frequently than desired site-specific mutation. Analysis of these particular mutation frequencies (Figure 2; Supplementary Tables S3–S7) demonstrated that the preferred indel sets are common for particular cleavage sites and independent of the cleavage system used, i.e., Cas9/sgRNA or PE2/pegRNA (compare Figure 2a,c,e,g,i with Figure 2b,d,f,h,j). The major advantage of the prime editor is an increased fidelity of genome editing, i.e., a higher ratio of the desired mutation over indels. In our experiments, we observed a number of editing sites where this is the case. For example, the position 3 application of PE2 leads to a 38:1 ratio of the desired mutation to indels (Figure 2f), while HR results in a 1.9:1 (Figure 2g) ratio, correspondingly. However, in several cases, such as positions 1, 2 and 5, editing fidelity is comparable for HR and PE2, with HR being slightly more precise (Figure 2). 

No dependency of the editing efficiency on the location of editing positions relative to the PAM was observed. Substitution of PAM in the case of position 4 might be expected to result in a complete loss of the possibility to re-cleave edited DNA sequences, and thus, decreased propensity of indel generation. However, the editing efficiency was very poor in this case, while the fidelity of editing, i.e., target mutation to indel ratio, was comparable to other editing sites.

**Figure 3:**
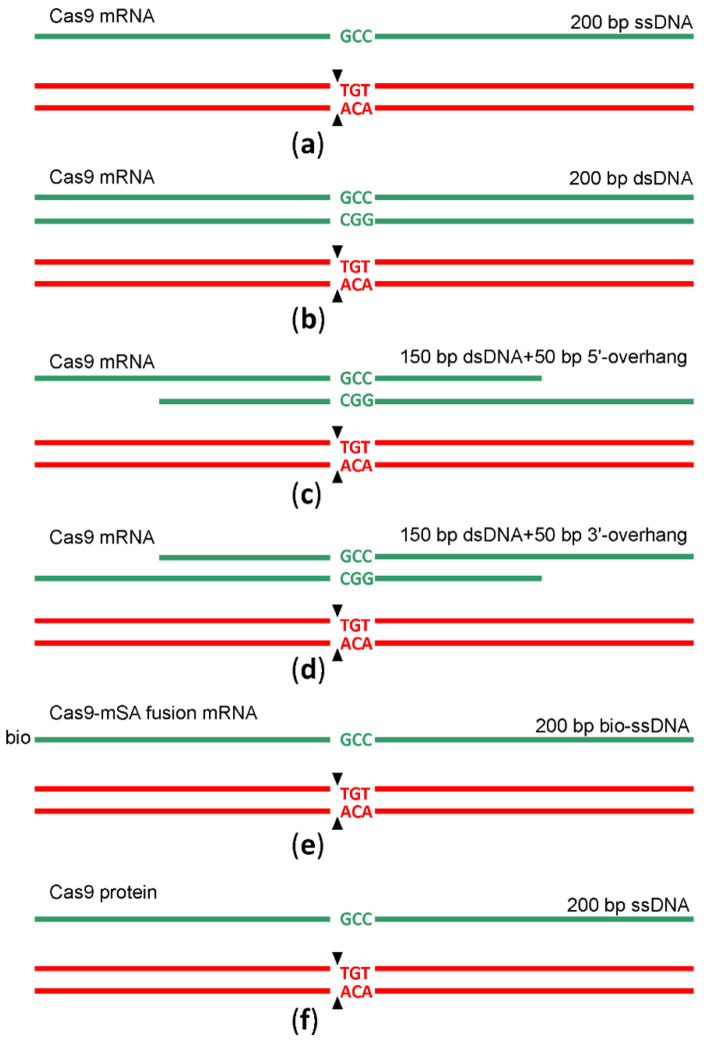
Scheme of genome editing by sgRNA guided cleavage and HR. The cleavage site is marked by black arrows. Red and green nucleotides mark the wild type and edited sequences. DNA templates used for HR are shown as green lines. (**a**) Scheme of genome editing by sgRNA directed cleavage and ssDNA guided HR; Cas9 was provided by injection of Cas9 mRNA. (**b**) Scheme of genome editing by sgRNA directed cleavage and dsDNA guided HR; Cas9 was provided by injection of Cas9 mRNA. (**c**) Scheme of genome editing by sgRNA directed cleavage and HR guided by DNA template with 5′-overhang; Cas9 was provided by injection of Cas9 mRNA. (**d**) Scheme of genome editing by sgRNA directed cleavage and HR guided by DNA template with 3′-overhang; Cas9 was provided by injection of Cas9 mRNA. (**e**) Scheme of genome editing by sgRNA directed cleavage and biotinylated ssDNA guided HR; Cas9-mSA was provided by injection of Cas9-mSA mRNA. (**f**) Scheme of genome editing by sgRNA directed cleavage and ssDNA guided HR; Cas9 was provided by injection of Cas9 protein.

**Figure 4:**
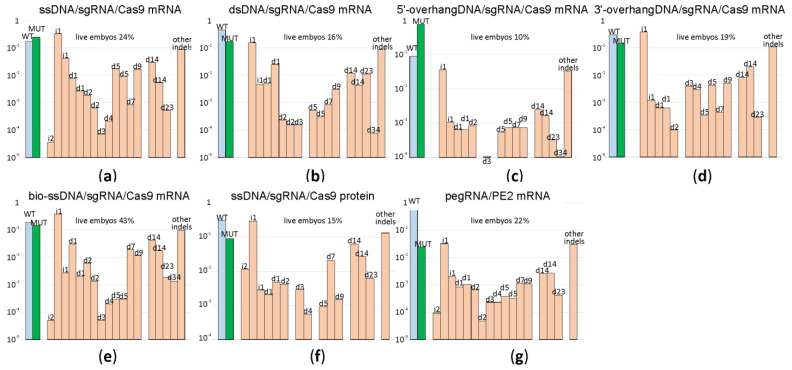
Comparison of genome editing efficiencies for HR programmed by different types of templates as well as alternative methods of genome editing. Shown are frequencies of allelic variants, in the logarithmic scale, detected in the blastocysts developed from the zygotes after microinjection. Blue bars correspond to the wild type genotype, green to the mutation programmed by a template, while pale red to insertions and deletions. Allelic variants are designated above the bars. (**a**) Frequency of genome editing products by sgRNA directed cleavage and ssDNA guided HR; Cas9 was provided by injection of Cas9 mRNA. (**b**) Frequency of genome editing products by sgRNA directed cleavage and dsDNA guided HR; Cas9 was provided by injection of Cas9 mRNA. (**c**) Frequency of genome editing products by sgRNA directed cleavage and HR guided by DNA template with 5′-overhang; Cas9 was provided by injection of Cas9 mRNA. (**d**) Frequency of genome editing products by sgRNA directed cleavage and HR guided by DNA template with 3′-overhang; Cas9 was provided by injection of Cas9 mRNA. (**e**) Frequency of genome editing products by sgRNA directed cleavage and biotinylated ssDNA guided HR; Cas9-mSA was provided by injection of Cas9-mSA mRNA. (**f**) Frequency of genome editing products by sgRNA directed cleavage and ssDNA guided HR; Cas9 was provided by injection of Cas9 protein. (**g**) Frequency of genome editing products by pegRNA directed cleavage and reverse transcription by PE2. For each construct, the percentage of zygotes developed to the blastocyst or morula stage is indicated above the graph.

In the natural way that double-strand DNA breaks repairs by HR [12], a double-stranded DNA, such as a sister chromatid or a homologous chromosome but not a single-stranded DNA, is used as a template. An obligatory step in HR is DNA 5′-end resection with the generation of the 3′-overhangs intruding into the intact dsDNA template. In a search for a better HR template for genome editing following Cas9 sgRNA guided DNA scission, we created a set of HR templates for a particular target region (chr5:66441024-6TGT/GCC). In addition to the 200 nt long ssDNA template, we used a blunt end dsDNA template of the same length and its variants with 50 nt long 3′ or 5′-overhangs (Figure 3, Supplementary Table S1) for microinjection into the mice zygotes along with Cas9 mRNA and sgRNA. To probe whether the substitution of Cas9 mRNA with Cas9 protein would increase the yield of editing, we appended our experiment with this composition. Additionally, aiming to increase the prevalence of HR over NHEJ, we used a recently suggested method [13] based on the application of a fusion between Cas9 and streptavidin (Cas9·mSA), which we utilized as the corresponding in vitro synthesized mRNA and sgRNA combined with the 5′-biotinylated ssDNA in the mixture for microinjection. After the microinjection of 1703 zygotes, 155 embryos developed to the blastocyst and 102 to morula stage. The survival rate for each microinjection experiment can be found in Supplementary Table S2. The survival of zygotes and successful development to morula and blastocyst stage after microinjection varied substantially across experiments, even for the same genome editing tool (Supplementary Table S2), likely due to experiment variability in the microinjection capillary opening diameter. On average, the survival was comparable for all genome editing tools (Figure 4), being somewhat better for mRNA Cas9·mSA/bio-ssDNA injection and somewhat worse for the mRNA Cas9/5′-overhanged DNA injection.

A subset of blastocysts (see Supplementary Table S2 for exact numbers) was used for genome DNA extraction, target DNA locus amplification and NGS analysis of the amplicons (Figure 4; Supplementary Figure S2).

The application of ssDNA, dsDNA templates as well as the templates with either 3′ or 5′ overhangs results in a nearly equivalent percentage of the desired mutation in the total products of double-strand break repair (Figure 4, Supplementary Figure S2). This is somewhat surprising since the 3′ overhanged DNA ends are natural intermediates of HR, which should be capable of annealing better with 3′-overhang HR templates. The experiment-to-experiment variability in the mutation yield is substantial (Figure 4, Supplementary Figure S2); for example, see the result of two replica experiments for the application of the 5′ overhang DNA template (Figure 4c, Supplementary Figure S2c). Usage of Cas9·mSA (Figure 4e) or Cas9 protein (Figure 4f) for microinjection did not resulted in an increase of the desired mutant genotype yield. As in the experiment with the editing of the five different genomic loci, the spectrum of indels is characteristic for the cleavage site and mildly dependent on the genome editing method used.

The fidelity of genome editing as a ratio between the frequency of desired mutation generation and indel by-products is comparable for all templates for the HR used (Figure 4).

## 3. Materials and Methods

For the editing of *M. musculus* genome, we amplified a fragment of pX458 plasmid (Addgene #48138) [14], coding the constant part of sgRNA appending it with T7 promoter and guide sequences (Figure 1, Supplementary Table S1). ssDNA templates, 200 nt long, were synthesized by IDT Inc. (Leuven, Belgium). dsDNA, 5′-overhanged and 3′-overhanged HR templates were obtained from the synthesized 200 nt oligonucleotides at 100 ng/μL final concentration by denaturation for 5 minutes at 96 °C and following slow cooling to a room temperature for annealing. A buffer 10 mM TrisCl pH 8, EDTA 0.1 mM was used for all microinjection solutions. For pegRNA [6] synthesis, we used PCR product of pX458 plasmid amplification with the primers containing T7 promoter and guide sequences as well as the reverse transcription template part as shown in Figure 1. The amplicon was used for in vitro transcription with a MEGAscript kit (Thermo Fisher scientific, Waltham, Massachusetts, USA). sgRNA purified with a QIAGEN (Venlo, The Netherlands) RNeasy MinElute CleanUp kit were mixed with mRNA coding for *S. pyogenesis* Cas9 (Thermo Fisher scientific, Waltham, Massachusetts, USA) to a final concentration 10 ng/μL sgRNA and 25 ng/μL Cas9 mRNA and 20 ng/μL DNA templates and used for murine zygotes microinjection. 

To generate PE2 and Cas9·mSA coding mRNAs, the plasmids pCMV-PE2 (Addgene #132775) [6] and PCS2+Cas9·mSA (Addgene #103882) [13] were used as templates for the amplification of parts corresponding to mRNAs with the addition of T7 promoter sequence to the 5′-end and A_50_ sequence to the 3′-end. An mMESSAGE mMACHINE kit (Thermo Fisher scientific, Waltham, Massachusetts, USA) was used for the generation of mRNAs by in vitro transcription. Cas9 protein (Thermo Fisher scientific, Waltham, Massachusetts, USA) 50 ng/μL f.c. was used instead of Cas9 coding mRNA where indicated. PE2 and Cas9·mSA coding mRNAs were used for microinjection at 25 ng/μL f.c. similar to that of Cas9 mRNA.

All manipulations were conducted in compliance with the protocol approved by the Local Bioethics Commission of the Research Center “Institute of Mitoengineering of Moscow State University” LLC, (Moscow, Russia) (http://www.vec-msu.ru/), Commission decision №79 dated July 2015. 

Mice C57BL/6J and CBA (Federal Research Center Institute of Cytology and Genetics, Siberian Branch, Russian Academy of Sciences) (ICG SB RAS) (Novosibirsk, Russia) were mated to obtain F1 hybrids C57BL/6J × CBA. The mice were kept in conditions free from pathogenic microorganisms, in individually ventilated cages (IVC system, TECNIPLAST S.p.A., Buguggiate, Italy), with free access to granulated autoclaved chow and reverse osmosis water; with light mode 12/12 (light on at 09:00); in rooms with an air exchange rate of at least 15 rev/h, with an air temperature of 20–24 °C, humidity 30–70%. The standard conditions of keeping in a barrier vivarium made it possible to stabilize the homeostasis and behavior of the animals.

Zygotes were obtained by the mating of the hybrid superovulated female mice with males using the standard procedure [1] briefly described below. The female mice, 1–2 months old, were subjected to superovulation via intraperitoneal administration of 100–140 μL (0.2 mg) of inhibin antiserum + 4 IU eCG (CARD HyperOva^®^, Cosmobio LTD, Tokyo, Japan) at 5 p.m., followed by an injection of 200 μL of human chorionic gonadotropin (hCG, 8 IU) (Chorulon^®^, MSD Animal Health, Kenilworth, N.J., USA) after 46 h, at 3 p.m. After the administration of hCG, the female mice were mated with males of the same genetic background. The mating was evaluated the following day at 10 a.m. based on the presence of vaginal plugs. Mice were sacrificed, their oviducts were removed, and zygotes were subsequently isolated into Flushing Media (CooperSurgical, Inc., Trumbull, CT, USA, #10845060). 

Isolated zygotes were immediately microinjected into the cytoplasm in the case of the PE2 editing and to a pronucleus in the case of the HR editing. Eppendorf FemtoJet 4i, Transferman 4r and Nikon Ti-E microscopes were used for microinjections. 

After microinjection, the zygotes were kept in 5% CO_2_ at 37 °C in the Sequential Fert medium (CooperSurgical, Inc., Trumbull, CT, USA, #83010010) for 2.5 days. The four to eight-cell embryos are transferred to the sequential blast medium (CooperSurgical, Inc., Trumbull, CT, USA, #83050010). After incubation for 1–2 days, the embryos successfully developed to the blastocyst stage were picked up, dissolved in 5 μL QuickExtract™ DNA Extraction Solution (Lucigen, Middleton, WI, USA) according to the manufacturer’s recommendations and used for amplification with the corresponding set of primers containing barcodes (Supplementary Table S1). In the first set of experiments, the following barcoded primers were used: POS1 HR (1_CHK_F1, 1_CHK_R), POS1 PE2 (1_CHK_F2, 1_CHK_R), POS2 HR (2_CHK_F1, 2_CHK_R), POS2 PE2 (2_CHK_F2, 2_CHK_R), POS3 HR (3_CHK_F, 3_CHK_R1), POS3 PE2 (3_CHK_F, 3_CHK_R2), POS4 HR (4_CHK_F1, 4_CHK_R), POS4 PE2 (4_CHK_F2, 4_CHK_R), POS5 HR (5_CHK_F, 5_CHK_R2), POS5 PE2 (5_CHK_F, 5_CHK_R1). For the analysis of editing efficiencies at position 5 with different HR templates, the following barcoded primers were used: POS5 PE2 (5_CHK_F, 5_CHK_R1), POS5 ssDNA (5_CHK_F, 5_CHK_R2), POS5 dsDNA (5_CHK_F, 5_CHK_R3), POS5 5′overhang (5_CHK_F, 5_CHK_R4). POS5 3′overhang (5_CHK_F, 5_CHK_R5), POS5 ssDNA and Cas9 protein (5_CHK_F, 5_CHK_R6), POS5 bio-ssDNA (5_CHK_F, 5_CHK_R7) (first experiment) and POS5 PE2 (5_CHK_F, 5_CHK_R5), POS5 ssDNA (5_CHK_F, 5_CHK_R2), POS5 dsDNA (5_CHK_F, 5_CHK_R1), POS5 5′overhang (5_CHK_F, 5_CHK_R4). POS5 3′overhang (5_CHK_F, 5_CHK_R3) (second experiment). Amplicons were pooled and subjected to NGS. Briefly, 100 ng of mixed PCR products was used as a DNA template to make a library with NEBNext^®^ Ultra™ II DNA Library Prep Kit for Illumina^®^ (NEB, Ipswich, MA, USA) according to the manual. The library was sequenced with at least 200 thousand single-end reads for every PCR product.

Reads were aligned to the amplicon sequence and classified into the wild type (WT), desired mutant programmed by the template (MUT) and different insertions and deletions (INDEL). We assumed equal amplification efficiency for all allelic variants and thus calculated relative editing efficiencies as a share of the corresponding mutation containing reads among total reads. Reads containing base substitutions other than programmed ones but not indels were presumed to be a result of PCR or sequencing errors. Potential PE2 reverse transcriptase errors were indistinguishable from the PCR and sequencing errors, and thus, not considered separately. Large DNA rearrangements and template multiplications could not be detected by the NGS of amplicons and thus could not be evaluated here.

## 4. Conclusions

(1) Application of novel methods of genome editing, such as PE2 or Cas9·mSA, do not increase the frequency of target mutation.
(2) Application of all types of templates for homology recombination, such as ssDNA, dsDNA, DNA duplexes with 3′ and 5′ overhangs, resulting in nearly equal frequency of the desired mutation.
(3) While the application of PE2 increases the fidelity of genome editing in a fraction of cases, generally, the fidelity of editing is comparable for PE2 directed editing and for Cas9-directed cleavage followed by HR with ssDNA, dsDNA, DNA duplexes with 3′ and 5′ overhangs as templates.

## Data Availability

Raw data have been submitted to the Sequence Read Archive under the accession number PRJNA765305.

## Supplementary Materials

The following are available online at https://www.mdpi.com/article/10.3390/ijms221910221/s1.
