# Supplementary material for: Comparative Analysis of Genome Editors Efficiency on a Model of Mice Zygotes Microinjection
Source: Int J Mol Sci. 2021 Sep 23;22(19):10221. doi: 10.3390/ijms221910221 (PMC8508852; doi:10.3390/ijms221910221)
Supplement: Supplementary file 1 [file ijms-22-10221-s001.zip › ijms-1318561-supplementary.pdf]

Supplementary information

Comparative analysis of genome editors efficiency on a model of mice zygotes microinjection

Olga A. Averina[1,2], Oleg A. Permyakov[2], Olga O. Grigorieva[2], Alexey S. Starshin[3], Alexander M. Mazur[3], Egor B. Prokhortchouk[3], Olga A. Dontsova[4,5,6] and Petr V. Sergiev[2,4,5*]

[1] Institute of functional genomics, , Lomonosov Moscow State University, Moscow, Russia; ifg.mouse@gmail.com
[2] Belozersky Institute of Physico-Chemical Biology, Lomonosov Moscow State University, Moscow, Russia; fxb@genebee.msu.ru
[3] Institute of Bioengineering, Research Center of Biotechnology of the Russian Academy of Sciences, Moscow, Russia; info@fbras.ru
[4] Department of chemistry, Lomonosov Moscow State University, Moscow, Russia; press@chem.msu.ru
[5] Center of life sciences, Skolkovo Institute of Science and Technology, Moscow, Russia; inbox@skoltech.ru
[6] Shemyakin-Ovchinnikov Institute of Bioorganic Chemistry, Moscow, Russia; office@ibch.ru
* Correspondence: Petr V. Sergiev; petya@genebee.msu.ru

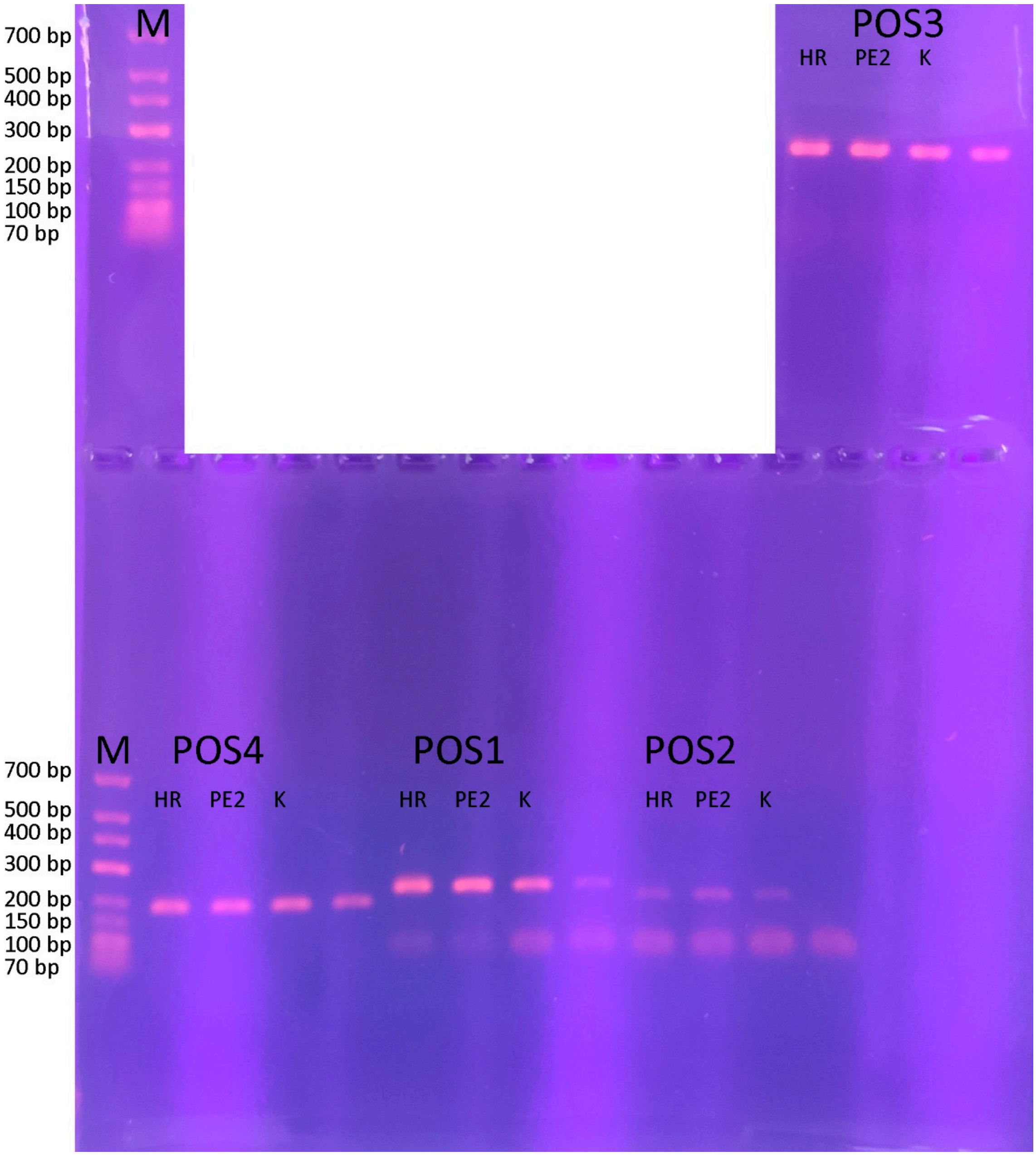

Supplementary Figure S1. Agarose gel of typical amplicons used for NGS analysis of editing efficiencies. Lanes are marked M (molecular weight markers); POS1-4 (amplicons corresponding to the editing positions 1-4); HR – amplicons derived from blastocysts developed form zygotes injected with Cas9 mRNA, sgRNA and ssDNA template for HR; PE2 – amplicons derived from blastocysts developed form zygotes injected with PE2 mRNA and pegRNA; K-wild type control.

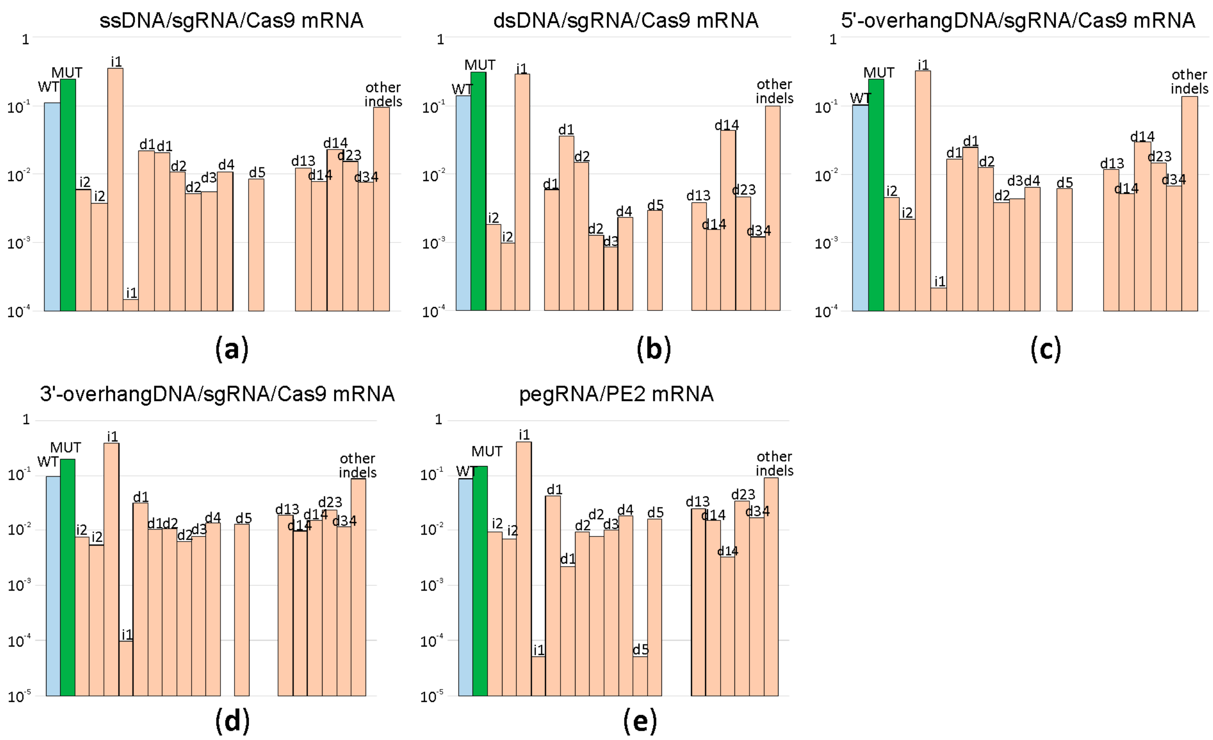

Supplementary Figure S2. Comparison of genome editing efficiencies for HR programmed by different types of templates as well as alternative methods of genome editing (the results of second experiment). Shown are frequencies of allelic variants, in the logarithmic scale, detected in the blastocysts developed from the zygotes after microinjection. Blue bars correspond to the wild type genotype, green to the mutation programmed by a template, while pale red to insertions and deletions. Allelic variants are designated above the bars. (**a**) Frequency of genome editing products by sgRNA directed cleavage and ssDNA guided HR; Cas9 was provided by injection of Cas9 mRNA. (**b**) Frequency of genome editing products by sgRNA directed cleavage and dsDNA guided HR; Cas9 was provided by injection of Cas9 mRNA. (**c**) Frequency of genome editing products by sgRNA directed cleavage and HR guided by DNA template with 5′-overhang; Cas9 was provided by injection of Cas9 mRNA. (**d**) Frequency of genome editing products by sgRNA directed cleavage and HR guided by DNA template with 3′-overhang; Cas9 was provided by injection of Cas9 mRNA. (**e**) Frequency of genome editing products by pegRNA directed cleavage and reverse transcription by PE2. For each construct the percentage of zygotes developed to the blastocyst or morula stage is indicated above the graph.

Supplementary Table S1. Sequences of primers.

CasSA_F	TGTAATACGACTCACTATAGGGAATACAAGCTACTTGTTCTTTTTGCA
CasSA_R	TTTTTTTTTTTTTTTTTTTTTTTTTTTTTTTTTTTTTTTTTTTTTTTTTTGCGAATTAAAAAACCTCCCACACC
T7 (PE_F)	TGTAATACGACTCACTATAG
PE_R	TTTTTTTTTTTTTTTTTTTTTTTTTTTTTTTTTTTTTTTTTTTTTTTTTTTGCGATGCAATTTTCTCATTTTATTAGGAAAGG
sgRNA rev	AAAAGCACCGACTCGGTGCC
1_HR	AGACCATCGAGACTGCCCTGGCTCAGTACCATCTCTCCCAGCTCTGCGTGGGCTTCAATGGGGGCAAGGACTGCACCGCACTCCTGCACCT CTTCCATGTAGCCGTGCAGAGGTAAGCCTACCCCTGGGAGCTAAGGCCTCAGAAAGACCCACCTGAAACCTCTCCAGCTCCCCACCCCAGA AAGCACAGCGTCAGCGTG
1_PEF	TGTAATACGACTCACTATAGGCTTACCTCTGCACGGCTGCAGTTTTAGAGCTAGAAATAGCAAG
1_PER	AAAACCTCTGCACGGCTACATGGAAGAGCACCGACTCGGTGCCACTTTTTC
1_CHK_F1	AGTCTCTGCGTGGGCTTCAATGG
1_CHK_F2	CTGCTCTGCGTGGGCTTCAATGG
1_CHK_R	GCTGATCACCTAGCTACCTTCC
2_HR	TGTGGCTGAGGACAGGTCTAGTGACTGATTGTCATTTCCCAGGACTGGACCTACAGAAACATCTGGGAGTTTCTGCGGCAGCTGTTTGTCC CATACTGCATCCTATATGACTGAGGGTAAGGTGGTTGACGGGCTGGGGTGAGAGGATGCTGGGAAAGAGAGCCCACCCATCACCCACAT CACCCACATGCTCCTGTCTC
2_PEF	TGTAATACGACTCACTATAGGACTGCATCCTATATGACAGAGTTTTAGAGCTAGAAATAGCAAG
2_PER	AAAACATCCTATATGACTGAGGGTAAGGCACCGACTCGGTGCCACTTTTTC
2_CHK_F1	AGTATCTGGGAGTTTCTGCGGC
2_CHK_F2	CTGATCTGGGAGTTTCTGCGGC
2_CHK_R	AGGGATGTGTACCTGGAGTGTT
3_HR	TTAAAAATATTGAACTTTTATTTTTTTACCATTAGAAAAATTCTTTAGAAACAGATATTCCTACTGTGAATATTTTAGCTGATGAAGAATTCCT TCCCCTCCAAGAAAACACGTTTGACCTGGTGGTTAGCAGCTTAAGGTTAGTAATCCATTTGTACTTTTAAAAAATATGTTACAGAGGCTAGT GAGATACTCTGACT
3_PEF	TGTAATACGACTCACTATAGGTCAAACGTGTTTTCTTGGAAGTTTTAGAGCTAGAAATAGCAAG
3_PER	AAAAACGTGTTTTCTTGGAGGGGAAGGGCACCGACTCGGTGCCACTTTTTC
3_CHK_F	AGCATTAGGAGTGTTCAACAATGA
3_CHK_R1	AGTGCTGCTAACCACCAGGTCAA
3_CHK_R2	CTGGCTGCTAACCACCAGGTCAA
4_HR	TGGATCATGACTTGATACTGAGCTGGGGGTATTTGCAGACTTGATACTGAGCTGGGGGTATTTGCAGTATTCTCTCAAACTGCCAGTAGTC TATTTTTAGGTATGTAAGAGAGCAACTGTTCTTGGAACAGAAAAGCCCTGCTGCACCGAGACACAATGCTGGCAGCTGCAGCGGTTTACA GAGGTGAGCCGCCCACTCC
4_PEF	TGTAATACGACTCACTATAGGAGTAGTCTATTTTTAGGTATGTTTTAGAGCTAGAAATAGCAAG
4_PER	AAAAGTCTATTTTTAGGTATGTAAGAGAGCAACTGCACCGACTCGGTGCCACTTTTTC
4_CHK_F1	AGTATTTGCAGTATTCTCTCAAACTGCC
4_CHK_F2	CTGATTTGCAGTATTCTCTCAAACTGCC
4_CHK_R	CCATCTGTGATTGCAGGTAGAA
5_HR	GTTTTTTCTTCTCAGATATTGAAATACTTCACGAAACATTTCTTAGCATTGAATCCAAAGATCACCGGTTACAGAATGTGAAGGTCATTCTGT TGCTACCTCGCGCCTCCAGCCTCGGTGTCAGCAACCCAGTGGAATTCATTCTAAATGAACACGAAGGTACTTGTTTTGATTTCTAAATTAAT GAAATTCATTGA
5_HR_DS	TAATCAATGAATTTCATTAATTTAGAAATCAAAACAAGTACCTTCGTGTTCATTTAGAATGAATTCCACTGGGTTGCTGACACCGAGGCTGG AGGCGCGAGGTAGCAACAGAATGACCTTCACATTCTGTAACCGGTGATCTTTGGATTCAATGCTAAGAAATGTTTCGTGAAGTATTTCAAT ATCTGAGAAGAAAAAAC
5_HR_5	ATTGTTTAATTTTGAAATCAATGAGTTTCATGATTAACTTCACGAGTAATTAATCAATGAATTTCATTAATTTAGAAATCAAAACAAGTACCT TCGTGTTCATTTAGAATGAATTCCACTGGGTTGCTGACACCGAGGCTGGAGGCGCGAGGTAGCAACAGAATGACCTTCACATTCTGTAACC GGTGATCTTTGGATTC
5_HR_3	CATTTAGAATGAATTCCACTGGGTTGCTGACACCGAGGCTGGAGGCGCGAGGTAGCAACAGAATGACCTTCACATTCTGTAACCGGTGAT CTTTGGATTCAATGCTAAGAAATGTTTCGTGAAGTATTTCAATATCTGAGAAGAAAAAACAGCAAGCAAACAAACAAAAGAAAACACTCGT GTTAAATATGGAAAATCTG
5_HR_bio	Biotin- GTTTTTTCTTCTCAGATATTGAAATACTTCACGAAACATTTCTTAGCATTGAATCCAAAGATCACCGGTTACAGAATGTGAAGGTC ATTCTGTTGCTACCTCGCGCCTCCAGCCTCGGTGTCAGCAACCCAGTGGAATTCATTCTAAATGAACACGAAGGTACTTGTTTTGATTTCTA AATTAATGAAATTCATTGA
5_PEF	TGTAATACGACTCACTATAGGTGGGTTGCTGACACCGAGGCGTTTTAGAGCTAGAAATAGCAAG
5_PER	C AAAATTGCTGACACCGAGGCTAGAGGCGCGAGGTAGCAACAGAAGCACCGACTCGGTGCCACTTTTT
5_ SGF	TGTAATACGACTCACTATAGGACACCGAGGCTGGAACAGCGGTTTTAGAGCTAGAAATAGCAAG
5_CHK_F	GTCAAGAGCTGCAGTCACAT
5_CHK_R1	AGTAGAATGAATTCCACTGGGTTGC
5_CHK_R2	CTGAGAATGAATTCCACTGGGTTGC
5_CHK_R3	TACAGAATGAATTCCACTGGGTTGC
5_CHK_R4	GCAAGAATGAATTCCACTGGGTTGC
5_CHK_R5	ACGAGAATGAATTCCACTGGGTTGC
5_CHK_R6	CATAGAATGAATTCCACTGGGTTGC
5_CHK_R7	TGAAGAATGAATTCCACTGGGTTGC
5_CHK_R8	GTCAGAATGAATTCCACTGGGTTGC

Supplementary Table S2. Survival rate after microinjection.

Construct	Injected zygotes	Developed to blastocyst stage	Developed to morula stage	Dead	Comments
**POS1 HR**	58	27 (47%)	4 (7%)	27 (47%)	
**POS1 PE2**	69	30 (43%)	0 (0%)	39 (57%)	
**POS2 HR**	182	14 (77%)	9 (5%)	159 (87%)	
**POS2 PE2**	62	18 (29%)	9 (15%)	35 (56%)	
	64	0 (0%)	0 (0%)	64 (100%)	Not analyzed
**POS3 HR**	153	26 (17%)	0 (0%)	127 (83%)	
**POS3 PE2**	132	12 (9%)	4 (3%)	116 (88%)	
**POS4 HR**	34	21 (62%)	6 (18%)	7 (20%)	
**POS4 PE2**	64	20 (31%)	0 (0%)	44 (69%)	
**POS5 ssDNA**	48	4 (8%)	13 (27%)	31 (65%)	
	49	5 (10%)	4 (8%)	40 (82%)	
	286	17 (6%)	28 (10%)	241 (84%)	
	57	11 (19%)	6 (11%)	40 (70%)	
	81	28 (35%)	17 (21%)	36 (44%)	
	134	0 (0%)	0 (0%)	134 (100%)	Not analyzed
	115	25 (22%)	15 (13%)	75 (65%)	Not analyzed
	40	13 (33%)	11 (28%)	16 (40%)	Not analyzed
**POS5 dsDNA**	55	8 (15%)	12 (22%)	35 (64%)	
	266	0 (0%)	0 (0%)	266 (100%)	Not analyzed
	88	21 (24%)	4 (5%)	63 (72%)	
	64	12 (19%)	20 (31%)	32 (50%)	
**POS5 5' overhang**	112	0 (0%)	0 (0%)	112 (100%)	Not analyzed
	92	0 (0%)	8 (9%)	84 (91%)	Not analyzed
	29	0 (0%)	0 (0%)	29 (100%)	Not analyzed
	104	21 (20%)	2 (2%)	81 (78%)	
	135	0 (0%)	0 (0%)	135 (100%)	Not analyzed
	135	0 (0%)	0 (0%)	135 (100%)	Not analyzed
	97	4 (4%)	34 (35%)	59 (61%)	
**POS5 3' overhang**	48	10 (21%)	5 (10%)	33 (69%)	
	38	5 (13%)	2 (5%)	31 (82%)	
	142	0 (0%)	0 (0%)	142 (100%)	Not analyzed
	34	9 (26%)	0 (0%)	25 (74%)	
	63	32 (51%)	0 (0%)	31 (49%)	Not analyzed
**POS5 5' bio-ssDNA**	63	16 (25%)	11 (17%)	26 (41%)	
**POS5 HR Cas9 protein**	138	17 (12%)	4 (3%)	117 (85%)	
**POS5 PE2**	83	6 (7%)	13 (16%)	64 (77%)	
	58	17 (29%)	3 (5%)	38 (66%)	
	97	48 (49%)	0 (0%)	49 (51%)	
	123	0 (0%)	0 (0%)	123 (100%)	Not analyzed
	76	4 (5%)	4 (5%)	68 (89%)	Not analyzed
**No injection**	10	10 (100%)	0 (0%)	0 (0%)	Not analyzed
	10	7 (70%)	3 (30%)	0 (0%)	Not analyzed
	10	6 (60%)	1 (10%)	3 (30%)	Not analyzed
	10	4 (40%)	0 (0%)	6 (60%)	Not analyzed
	10	10 (100%)	0 (0%)	0 (0%)	Not analyzed
	10	7 (70%)	0 (0%)	3 (30%)	Not analyzed
	10	7 (70%)	0 (0%)	3 (30%)	Not analyzed
	10	7 (70%)	0 (0%)	3 (30%)	Not analyzed
	14	6 (43%)	0 (0%)	8 (57%)	Not analyzed
	10	4 (40%)	1 (10%)	5 (50%)	Not analyzed
	10	2 (20%)	3 (30%)	5 (50%)	Not analyzed
	10	2 (20%)	3 (30%)	5 (50%)	Not analyzed
	10	3 (30%)	6 (60%)	1 (10%)	Not analyzed
	10	4 (40%)	2 (20%)	4 (40%)	Not analyzed
	10	7 (70%)	0 (0%)	3 (30%)	Not analyzed
	10	0 (0%)	2 (20%)	8 (80%)	Not analyzed
	10	3 (30%)	0 (0%)	7 (70%)	Not analyzed
	10	5 (50%)	0 (0%)	5 (50%)	Not analyzed
	10	5 (50%)	0 (0%)	5 (50%)	Not analyzed
	10	5 (50%)	1 (10%)	4 (40%)	Not analyzed
	10	6 (60%)	0 (0%)	4 (40%)	Not analyzed
	10	5 (50%)	0 (0%)	5 (50%)	Not analyzed
	10	4 (40%)	2 (20%)	4 (40%)	Not analyzed
	10	7 (70%)	3 (30%)	0 (0%)	Not analyzed
	10	4 (40%)	3 (30%)	3 (30%)	Not analyzed
	10	5 (50%)	5 (50%)	0 (0%)	Not analyzed
	10	4 (40%)	2 (20%)	4 (40%)	Not analyzed
	10	5 (50%)	2 (20%)	3 (30%)	Not analyzed
	10	6 (60%)	3 (30%)	1 (10%)	Not analyzed
	10	7 (70%)	0 (0%)	3 (30%)	Not analyzed
	8	1 (13%)	0 (0%)	9 (113%)	Not analyzed
	10	9 (90%)	0 (0%)	1 (10%)	Not analyzed
	48	24 (50%)	20 (42%)	4 (8%)	Not analyzed
	26	12 (46%)	11 (42%)	3 (12%)	Not analyzed

Supplementary Table S3. Sequences and frequencies of allelic variants at position 1.

	Allelic variant	Editing strategy/ frequency of allelic variant	
		HR	PE2
**WT**	CTCTGCGTGGGCTTCAATGGGGGCAAGGACTGCACCGCACTCCTGCACCTCTTCCATG CAGCCGTGCAGAGGTAAGC	0,26	0,96
**MUT**	CTCTGCGTGGGCTTCAATGGGGGCAAGGACTGCACCGCACTCCTGCACCTCTTCCATG TAGCCGTGCAGAGGTAAGC	0,067	0,002
**i1**	CTCTGCGTGGGCTTCAATGGGGGCAAGGACTGCACCGCACTCCTGCACCTCTTCCATGC **A**AGCCGTGCAGAGGTAAGC	0,28	0,006
**d3**	CTCTGCGTGGGCTTCAATGGGGGCAAGGACTGCACCGCACTCCTGCACCTCTTCCATGC ···CGTGCAGAGGTAAGC	0,076	0,0015
**d8**	CTCTGCGTGGGCTTCAATGGGGGCAAGGACTGCACCGCACTCCTGCACCTCTTCCATG ········CAGAGGTAAGC	0,026	0,0005
**d3**	CTCTGCGTGGGCTTCAATGGGGGCAAGGACTGCACCGCACTCCTGCACCTCTTCCTAC C CCTGGGAGC···TAAGC	0,019	0,0002
**d8**	CTCTGCGTGGGCTTCAATGGGGGCAAGGACTGCACCGCACTCCTGCACCTCTTCC··· ·····GTGCAGAGGTAAGC	0,017	1E-04
**d1**	CTCTGCGTGGGCTTCAATGGGGGCAAGGACTGCACCGCACTCCTGCACCTCTTCCATG T GCCGTGCAGAGGTAAGC	0,01	0,0002
**i2**	CTCTGCGTGGGCTTCAATGGGGGCAAGGACTGCACCGCACTCCTGCACCTCTTCCATG**CCAT**GCCGTGCAGAGGTAAGC	0,01	0,0002
**d4**	CTCTGCGTGGGCTTCAATGGGGGCAAGGACTGCACCGCACTCCTGCACCTCTTCCATGC ····GTGCAGAGGTAAGC	0,007	0,00015
**d2**	CTCTGCGTGGGCTTCAATGGGGGCAAGGACTGCACCGCACTCCTGCACCTCTTCCATGG ··CCGTGCAGAGGTAAGC	0,006	0,0002

d66	CTCTGCGTGGGCTTCAATGGGGGCAAGGACTGCACCGCACTCCTGCACCTCTTCCATG (d66)CAGCTCCCCACCCC	0,009	0,0002
i2	CTCTGCGTGGGCTTCAATGGGGGCAAGGACTGCACCGCACTCCTGCACCTCTTCCATG**CCAT**GCCGTGCAGAGGTAAGC	0,006	1E-04
i1	CTCTGCGTGGGCTTCAATGGGGGCAAGGACTGCACCGCACTCCTGCACCTCTTCCATG **T**AAGCCGTGCAGAGGTAAGC	0,006	1E-04
d22	CTCTGCGTGGGCTTCAATGGGGGCAAGGACTGCACCGCACTCCTGCACCTCTTCCATG (d22)CCCCTGGGAGCTAA	0,005	0,0001
d9	CTCTGCGTGGGCTTCAATGGGGGCAAGGACTGCACCGCACTCCTGCACCTCTTC···· ·····GTGCAGAGGTAAGC	0,005	3E-05
d1	CTCTGCGTGGGCTTCAATGGGGGCAAGGACTGCACCGCACTCCTGCACCTCTTCCATG C·GCCGTGCAGAGGTAAGC	0,005	7E-05

Supplementary Table S4. Sequences and frequencies of allelic variants at position 2.

	Allelic variant	Editing strategy/ frequency of allelic variant	
		HR	PE2
WT	ATCTGGGAGTTTCTGCGGCAGCTGTTTGTCCCATACTGCATCCTATATGAC AGAGGGTAAGGTGGTTGACGGGCTGG	0,68	0,97
MUT	ATCTGGGAGTTTCTGCGGCAGCTGTTTGTCCCATACTGCATCCTATATGAC TGAGGGTAAGGTGGTTGACGGGCTGG	0,1	0,005
d4	ATCTGGGAGTTTCTGCGGCAGCTGTTTGTCCCATACTGCATCCTATATGA· ···GGGTAAGGTGGTTGACGGGCTGG	0,05	0,0007
d3	ATCTGGGAGTTTCTGCGGCAGCTGTTTGTCCCATACTGCATCCTATATGA· ·GA·GGTAAGGTGGTTGACGGGCTGG	0,02	0,0004
d8	ATCTGGGAGTTTCTGCGGCAGCTGTTTGTCCCATACTGCATCCTAT····· ···GGGTAAGGTGGTTGACGGGCTGG	0,014	0,00015
i1	ATCTGGGAGTTTCTGCGGCAGCTGTTTGTCCCATACTGCATCCTATATGAC**C**AGAGGGTAAGGTGGTTGACGGGCTGG	0,013	0,0002
d9	ATCTGGGAGTTTCTGCGGCAGCTGTTTGTCCCATACTGCATCCTA······ ···GGGTAAGGTGGTTGACGGGCTGG	0,005	2,5E-05
d3	ATCTGGGAGTTTCTGCGGCAGCTGTTTGTCCCATACTGCATCCTATATAGA ···GGGTAAGGTGGTTGACGGGCTGG	0,011	0,0002
d5	ATCTGGGAGTTTCTGCGGCAGCTGTTTGTCCCATACTGCATCCTATA···· ·GAGGGTAAGGTGGTTGACGGGCTGG	0,008	0,0001
d14	ATCTGGGAGTTTCTGCGGCAGCTGTTTGTCC············TATA··TG AGAGGGTAAGGTGGTTGACGGGCTGG	0,007	1E-04
d1	ATCTGGGAGTTTCTGCGGCAGCTGTTTGTCCCATACTGCATCCTATATGAC ·GAGGGTAAGGTGGTTGACGGGCTGG	0,005658	6,24E-05
d12	ATCTGGGAGTTTCTGCGGCAGCTGTTTGTCCCATACTGCA··········· ·GAGGGTAAGGTGGTTGACGGGCTGG	0,005606	7,49E-05
d15	ATCTGGGAGTTTCTGCGGCAGCTGTTTGTCCCATACTG············· ··AGGGTAAGGTGGTTGACGGGCTGG	0,00313	6,24E-05

Supplementary Table S5. Sequences and frequencies of allelic variants at position 3.

	Allelic variant	Editing strategy/ frequency of allelic variant	
		HR	PE2
WT	GCTGCTAACCACCAGGTCAAACGTGTTTTCTTGGAAGGGAAGGAATTCTTCATCAGCTAAAATATTCACAGTAGGAA	0,98	0,99
MUT	GCTGCTAACCACCAGGTCAAACGTGTTTTCTTGGAGGGGAAGGAATTCTTCATCAGCTAAAATATTCACAGTAGGAA	0,013	0,005
d1	GCTGCTAACCACCAGGTCAAACGTGTTTTCTTG·AAGGGAAGGAATTCTTCATCAGCTAAAATATTCACAGTAGGAA	0,007	0,0001

Supplementary Table S6. Sequences and frequencies of allelic variants at position 4.

	Allelic variant	Editing strategy/ frequency of allelic variant	
		HR	PE2
WT	ATTTGCAGTATTCTCTCAAACTGCCAGTAGTCTATTTTTAGGT ATGGGTGAGAGCAACTGTTCTTGGAA	0,97	0,9995
MUT	ATTTGCAGTATTCTCTCAAACTGCCAGTAGTCTATTTTTAGGT ATGTAAGAGAGCAACTGTTCTTGGAA	0,015	0,0003
d2	ATTTGCAGTATTCTCTCAAACTGCCAGTAGTCTATTTTTAGGT ··GGGTGAGAGCAACTGTTCTTGGAA	0,005	0,0001
d12	ATTTGCAGTATTCTCTCAAACTGCCAGTAG············ TATGGGTGAGAGCAACTGTTCTTGGAA	0,003	4E-05
i11	ATTTGCAGTATTCTCTCAAACTGCCAGTAGTCTATTTTTAGGT**GAGTAGTCTAT**TATGGGTGAGAGCAACTGTTCTTGGAA	0,003	1E-05

Supplementary Table S7. Sequences and frequencies of allelic variants at position 5.

	Allelic variant	Editing strategy/ frequency of allelic variant											
		ssDNA	ssDNA	dsDNA	dsDNA	5' over	5' over	3' over	3' over	bio-ssDNA	Cas9 prot	PE2	PE2
WT	AGAATGAATTCCACTGGGTTGCTGACACCGAGGCTGGAACA GCGAGGTAGCAACAGAATGACCTTCACATTCTGTA	0,18	0,11	0,46	0,14	0,09	0,10	0,30	0,10	0,19	0,31	0,84	0,09
MUT	AGAATGAATTCCACTGGGTTGCTGACACCGAGGCTGGAGGC GCGAGGTAGCAACAGAATGACCTTCACATTCTGTA	0,25	0,24	0,17	0,31	0,78	0,25	0,14	0,20	0,15	0,09	0,02	0,15
i2	AGAATGAATTCCACTGGGTTGCTGACACCGAGGCTGGAACA**TA**GCGAGGTAGCAACAGAATGACCTTCACATTCTGTA	0	0,006	0	0,002	0	0,005	0	0,008	0	0	0	0,009
i2b	AGAATGAATTCCACTGGGTTGCTGACACCGAGGCTGGAACA**AA**GCGAGGTAGCAACAGAATGACCTTCACATTCTGTA	3,5E-05	0,004	0	0,001	0	0,002	0	0,005	5E-05	0,01	9E-05	0,007
i1	AGAATGAATTCCACTGGGTTGCTGACACCGAGGCTGGAACA**A** GCGAGGTAGCAACAGAATGACCTTCACATTCTGTA	0,33	0,36	0,16	0,29	0,04	0,32	0,37	0,40	0,39	0,29	0,03	0,42
i1b	AGAATGAATTCCACTGGGTTGCTGACACCGAGGCTGGAAC**T**A GCGAGGTAGCAACAGAATGACCTTCACATTCTGTA	0,04	0,00015	0,005	0	0,001	0,0002	0,001	1E-04	0,003	0,003	0,002	5E-05
d1	AGAATGAATTCCACTGGGTTGCTGACACCGAGGCTGGAA·A GCGAGGTAGCAACAGAATGACCTTCACATTCTGTA	0,008	0,022	0,005	0,006	0,0006	0,017	0,0006	0,03	0,03	0,002	0,0008	0,04
d1b	AGAATGAATTCCACTGGGTTGCTGACACCGAGGCTGGAAC· GCGAGGTAGCAACAGAATGACCTTCACATTCTGTA	0,003	0,02	0,025	0,036	0,0006	0,025	0,0006	0,01	0,002	0,005	0,001	0,002
d2	AGAATGAATTCCACTGGGTTGCTGACACCGAGGCTGGAA·· GCGAGGTAGCAACAGAATGACCTTCACATTCTGTA	0,002	0,011	0,0002	0,015	0,0008	0,013	1E-04	0,011	0,006	0,004	0,0007	0,01
d2	AGAATGAATTCCACTGGGTTGCTGACACCGAGGCTGGAG·· GCGAGGTAGCAACAGAATGACCTTCACATTCTGTA	0,0006	0,005	0,0002	0,001	0	0,004	0	0,006	0,001	0	5E-05	0,008
d3	AGAATGAATTCCACTGGGTTGCTGACACCGAGGCTGGAAC· ··GAGGTAGCAACAGAATGACCTTCACATTCTGTA	7E-05	0,006	0,0002	0,0009	0,0001	0,004	0,004	0,008	5E-05	0,003	0,0002	0,01
d4	AGAATGAATTCCACTGGGTTGCTGACACCGAGGCTGG···· GCGAGGTAGCAACAGAATGACCTTCACATTCTGTA	0,0002	0,011	0	0,002	0	0,007	0,003	0,014	0,0002	0,0006	0,0002	0,018
d5	AGAATGAATTCCACTGGGTTGCTGACACCGAGGCTGGA··· ··GAGGTAGCAACAGAATGACCTTCACATTCTGTA	0,017	0	0,0005	0	0,0005	0	0,0003	0	0,0003	0	0,0004	5E-05
d5b	AGAATGAATTCCACTGGGTTGCTGACACCGAGGCTGG···· ·CGAGGTAGCAACAGAATGACCTTCACATTCTGTA	0,009	0,009	0,0003	0,003	0,0007	0,006	0,004	0,013	0,0003	0,0009	0,0003	0,016
d7	AGAATGAATTCCACTGGGTTGCTGACACCGAGGCTGGA·· ·····GGTAGCAACAGAATGACCTTCACATTCTGTA	0,0009	0	0,0008	0	0,0007	0	0,0004	0	0,02	0,021	0,001	0
d9	AGAATGAATTCCACTGGGTTGCTGACACCGAGGC······ ···GAGGTAGCAACAGAATGACCTTCACATTCTGTA	0,017	0	0,003	0	0,0007	0	0,005	0	0,011	0,001	0,001	0
d13	AGAATGAATTCCACTGGGTTGCTGACACCGAGGCT······ ·······AGCAACAGAATGACCTTCACATTCTGTA	0	0,012	0	0,004	0	0,012	0	0,019	0	0	0	0,024
d14	AGAATGAATTCCACTGGGTTGCTGACACCGAGGTAG····· ·········CAACAGAATGACCTTCACATTCTGTA	0,03	0,008	0,012	0,002	0,003	0,005	0,008	0,01	0,043	0,062	0,003	0,015
d14b	AGAATGAATTCCACTGGGTTGCTGACACCGAGGCTGGAACA ·············GAATGACCTTCACATTCTGTA	0,005	0,023	0,004	0,04	0,002	0,03	0,02	0,015	0,018	0,027	0,003	0,003
d23	AGAATGAATTCCACTGGGTTGCTGACACCGAGGCTGGAC·· ·····················CTTCACATTCTGTA	0,0005	0,015	0,011	0,005	0,0003	0,015	0,0003	0,023	0,002	0,006	0,0004	0,034
d34	AGAATGAATTCCACTGGGTTGCTGACACC············ ·····················TTCACATTCTGTA	0	0,008	8E-05	0,001	0,0001	0,007	0	0,012	0,001	0	0	0,017
